# *Ageratum conyzoides* Extract Ameliorates Testosterone-Induced Benign Prostatic Hyperplasia via Inhibiting Proliferation, Inflammation of Prostates, and Induction of Apoptosis in Rats

**DOI:** 10.3390/nu16142267

**Published:** 2024-07-14

**Authors:** Eun-Hye Chung, Jeong-Won Kim, Jin-Hwa Kim, Ji-Soo Jeong, Jong-Hwan Lim, So-Young Boo, Je-Won Ko, Tae-Won Kim

**Affiliations:** 1 BK21 FOUR Program, College of Veterinary Medicine, Chungnam National University, 99 Daehak-ro, Daejeon 34131, Republic of Korea; ksissb1293@gmail.com (E.-H.C.); lilflflb@gmail.com (J.-W.K.); jinhwa926@g.cnu.ac.kr (J.-H.K.); jisooj9543@gmail.com (J.-S.J.); labong1966@gmail.com (S.-Y.B.); 2 EveryH Co., Ltd., Seoul 06103, Republic of Korea; jonghwan.lim@everyh.co.kr

**Keywords:** benign prostatic hyperplasia, *Ageratum conyzoides*, rat model, androgen receptor, inflammation

## Abstract

*Ageratum conyzoides*, an annual herbaceous plant that inhabits tropical and subtropical regions, has been traditionally used in Asia, Africa, and South America for phytotherapy to treat infectious and inflammatory conditions. However, the pharmacological effects of standardized ethanolic extract of *Ageratum conyzoides* (ACE) on benign prostatic hyperplasia (BPH) remain unexplored. The objective of this research is to examine the potential physiological impacts of ACE, a traditionally utilized remedy for inflammatory ailments, in a rat model with BPH induced by testosterone propionate (TP). Rats were subcutaneously administered TP (3 mg/kg) to induce BPH and concurrently orally administered ACE (20, 50, and 100 mg/kg) daily for 42 days. ACE markedly improved BPH characteristics, including prostate weight, prostate index, and epithelial thickness, while also suppressing androgens and related hormones. The findings were supported by a decrease in androgen receptor and downstream signals associated with BPH in the prostate tissues of the ACE groups. Furthermore, increased apoptotic signals were observed in the prostate tissue of the ACE groups, along with heightened detection of the apoptotic nucleus compared to the BPH alone group. These changes seen in the group that received finasteride were similar to those observed in this group. These findings suggest that ACE shows promise as an alternative phytotherapeutic agent for treating BPH.

## 1. Introduction

Owing to the extended average lifespan, there is a growing occurrence of age-associated conditions, including cancers, neurodegenerative diseases, cardiovascular problems, and reproductive dysfunctions [1]. Benign prostatic hyperplasia (BPH) is a prevalent, age-related male reproductive condition characterized by a non-cancerous growth of the prostate gland. The incidence of BPH among men aged 40 is approximately 20%, with prevalence increasing to 70% by the age of 60 [2]. BPH is identified by the increase in size of the prostate gland, which causes compression of the urethra and disruption of urinary flow. This results in the development of symptoms related to lower urinary tract dysfunction (LUTS) including hematuria, nocturia, prolonged micturition, and incomplete bladder emptying. These symptoms hamper the overall well-being in men, and the persistence of LUTS can result in complications such as urinary system infections [3].

Although the etiology of BPH is unclear, several factors such as androgens, inflammation, cell proliferation factors, and decreased apoptosis in the prostate gland are involved in BPH progression [4,5,6]. Specifically, the role of male sex hormones, testosterone, and dihydrotestosterone (DHT), has been claimed significantly [3,7]. DHT is the most influential androgenic hormone associated with BPH progression. DHT is synthesized by the enzymatic conversion of testosterone to DHT by the enzyme 5α-reductase, whose activity increases with aging. Testosterone and DHT both compete for binding to the androgen receptor (AR), which subsequently translocates to the nucleus to activate androgen-regulated pathways involved in cell proliferation and differentiation. DHT exhibits greater intrinsic activity and affinity for the AR compared to testosterone [8].

Inflammation may also be a risk factor for BPH development. Prostrate inflammation is related to its growth in pathological changes, such as BPH and prostatic cancers [9]. In particular, the prostaglandin pathway, which involves cyclooxygenase-2 (COX-2) and prostaglandin E2 (PGE2), contributes to uncontrolled cell proliferation [10,11]. Moreover, this pathway might be involved in upregulating aromatase, which converts testosterone to estradiol, which in turn activates estrogen receptor α (ERα), a transcription factor in the progression of prostatic proliferation [12].

Phytotherapy has recently been suggested as a treatment for BPH due to its well tolerance and lower side effects [13]. These medicinal plants contain various polyphenols, which have been reported to suppress BPH through their anti-inflammatory and antioxidant properties and the inhibition of molecules associated with cell survival [14]. *Ageratum conyzoides* (*A. conyzoides*), belonging to the *Asteraceae* family, is a flowering species found in tropical and subtropical regions [15]. *A. conyzoides* is traditionally used as herbal therapy in Asia, Africa, and South America due to its antimicrobial, antifungal, anti-inflammatory, and wound-healing properties [16]. *A. conyzoides* contains many phytochemical compounds, including alkaloids, flavonoids, and terpenes [15]. In clinical trials, the therapeutic potential of A. conyzoides for male reproductive disease was demonstrated. It was found to inhibit the 5α-reductase gene expression in human prostate cells and mitigate symptoms related to BPH [17]. Additionally, *A. conyzoides* extract (ACE) improved BPH conditions in a BPH mouse model [18]. Although the pharmacological effects of various ACEs have been reported, the pharmaceutical efficacy of standardized ACE against BPH and its associated mechanisms have not been fully elucidated. Hence, the objective of this study was to assess the therapeutic efficacy of ACE in a rat model of benign prostatic hyperplasia induced by TP and to investigate the underlying mechanisms involved in the progression of BPH.

## 2. Materials and Methods

### 2.1. Preparation of ACE (AGEprost^®^) and Phytochemical Analysis

The standardized ACE (AGEprost^®^), obtained by hydroethanol extraction of its aerial parts, was provided by Gencor Pacific Inc. in Hong Kong. The dried *A. conyzoides* aerial parts were extracted with 90% ethanol. Dried aerial parts of *Ageratum conyzoides* are loaded into a stainless-steel reactor together with ethyl alcohol and water. Extraction is achieved by heating the mixture under gentle pressure in a closed system, where the extract is repeatedly pumped back to the herb bed. This process is repeated until the desired extraction is obtained, after which the mixture is filtered. The extracts are distilled partly under normal conditions without vacuum. The distillation is continued under vacuum to remove the solvent. The extract is allowed to stand to precipitate the residues at room temperature. This step ensures the total removal of any alkaloids in the extract. It is filtered and then transferred to a drying unit to obtain the product in powder form. This powder is further processed in a Multimill to obtain a fine mesh size. Finally, it is then sieved using a Sifter to ensure consistent particle size. It is blended with 5% maltodextrin to make it uniform and homogenous and it is heat-treated and sieved again.

The phytochemical composition of ACE was analyzed using high-performance liquid chromatography (HPLC) with an ultraviolet detector (Shimadzu, Tokyo, Japan). The analysis was performed on a Sunfire C18 column (4.6 × 250 mm, 5 μm; Waters, MA, USA) at 35 °C. The mobile phase consisted of 0.1% formic acid dissolved in distilled water (A) and acetonitrile (B), with a flow rate of 0.8 mL/min, and the gradient conditions were set as follows: 5% B, 1–5 min; 5–95% B, 5–25 min; 95–95% B, 25–30 min; 95–5% B, 30–35 min; 5–5% B, 35–45 min. The detection wavelength was 330 nm, and the injection volume was 10 μL. Reference standards for 5′-methoxynobiletin (≥98% purity; Chemfaces, Wuhan, China) were used to determine its content in standardized ACE (AGEprost^®^). The retention time of 5′-methoxynobiletin was 23.8 min and the contents were determined to be approximately 0.4% of 5′-methoxynobiletin as shown in Supplementary Figure S1.

### 2.2. Animals

Male Sprague–Dawley rats (8-week-old, initial body weight = 267.4 ± 10.9 g) were purchased from SAMTAKO (Osan, Republic of Korea). Prior to the experiment, the rats underwent a period of acclimation lasting a minimum of seven days, during which they were subjected to standard environmental conditions including a humidity range of 50 ± 5%, a temperature range of 22 ± 3 °C, a 12 h light–dark cycle from 7 am to 7 pm, and an airflow rate of 15 to 20 air changes per hour. The animal experiments conducted adhered to the regulations set forth by the Institutional Animal Care and Use Committee of Chungnam National University (approval no. 202309A-CNU-157).

### 2.3. TP Induced Rat BPH Model

BPH in rats was induced following the method described previously [19]. Testosterone propionate (TP; Tokyo Chemical Ins. Co., Tokyo, Japan) dissolved in corn oil (3 mg/kg) was administered daily via subcutaneous injection (S.C.) to the animals, excluding the normal control group, for six weeks. The rats were randomly assigned to six groups as follows (*n* = 5/group): (a) normal control (NC, corn oil S.C. + phosphate buffered saline (PBS) orally (P.O.)) group; (b) BPH (TP 3 mg/kg/day S.C. + PBS P.O.) group; (c) Fina (positive control; TP 3 mg/kg/day S.C. + Finasteride 10 mg/kg/day P.O.) group; and (d–f) ACE (TP 3 mg/kg/day S.C. + 20 mg, 50 mg, 100 mg/kg/day ACE P.O., respectively) groups. The 5α-reductase 2 inhibitor, finasteride, was obtained from Tokyo Chemical Industry Co., Ltd. The materials mentioned above were orally administered on a daily basis for a duration of six weeks. The rats were weighed every week to determine the dosage. Following the last treatment, the animals underwent a 12 h fasting period before being euthanized, at which point whole blood samples were collected from the cauda vena cava. The entire prostate was promptly excised and weighed, and the prostate index was calculated by determining the ratio of prostate weight (mg) to body weight (100 g).

### 2.4. Quantification of Androgen-Related Factors in Plasma and Prostate Tissue

The plasma and prostate levels of testosterone (MyBioSource Inc., San Diego, CA, USA), DHT (MyBioSource Inc., San Diego, CA, USA), 5α-reductase 1/2 (Cusabio Biotech, Wuhan, China), and prostate-specific antigen (PSA, MyBioSource Inc., San Diego, CA, USA) were measured using a commercial enzyme-linked immunosorbent assay (ELISA) kit, following the manufacturer’s instructions. The levels of aspartate aminotransferase, alanine aminotransferase, blood urea nitrogen, creatinine, albumin, triglycerides, and total cholesterol were measured using Fuji Dry-Chem slides (Fuji Film Co., Tokyo, Japan).

### 2.5. Histopathological Examination

Prostatic tissue samples were immersed in a 10% formalin solution for fixation, followed by embedding in paraffin. Subsequently, sections measuring 4 μm in thickness were prepared and subjected to staining using hematoxylin and eosin (H&E) from Biorad, Ca, USA. The thickness of the prostate epithelium was measured using ImageJ software (version 1.8.0_172, National Institutes of Health, Bethesda, MD, USA) across five randomly selected areas for each group at 400× magnification.

### 2.6. Terminal Deoxynucleotidyl Transferase Fluorescein-dUTP Nick End-Labeling (TUNEL) Staining

Prostate tissues that had been deparaffinized and dehydrated were cut into 4 μm sections and subjected to treatment with citric buffer (pH 6.0) for 5 min at 121 °C. The sections were subsequently exposed to hydrogen peroxide diluted in distilled water (3%) for 5 min. To detect apoptotic cells, the TUNEL assay kit (Abcam, Cambridge, UK) was utilized in accordance with the provided guidelines. After counterstaining with hematoxylin and mounting with a coverslip, the samples were examined under a microscope at various magnifications. Apoptotic cells, characterized by DNA fragmentation in late apoptosis, were assessed in five randomly chosen microscopic fields at a magnification of 400×. The quantification of apoptotic cells was performed using ImageJ software (version 1.8.0_172, National Institutes of Health, Bethesda, MD, USA).

### 2.7. Immunoblotting

Rat prostate tissues were homogenized with a cold tissue lysis/extraction reagent (Sigma–Aldrich Co., St. Louis, MO, USA), and protein concentrations were determined using the Bradford reagent (Bio-Rad Laboratories, Watford, Herts, UK). Equal quantities of proteins were applied to sodium dodecyl sulfate-polyacrylamide gels and transferred to polyvinylidene difluoride membranes (Millipore Corp., Bedford, MA, USA). Membranes were blocked using 5% skimmed milk in tris-buffered saline containing 0.1% Tween-20 (TBS-T) for 1 h at 23 °C and then reacted with primary antibodies (1:1000) at 4 °C overnight. The following antibodies were used in this research: 5α-reductase 1 and 5α-reductase 2 were obtained from Invitrogen Life technologies (Carlsbad, CA, USA) and steroid receptor coactivator 1 (SRC-1), proliferating cell nuclear antigen (PCNA), PSA, COX-2, aromatase, AR, Bcl-2-associated X protein (Bax), B-cell lymphoma 2 (Bcl-2), cleaved caspase-9 (Cas-9), Cas-3, cleaved poly (ADP-ribose) polymerase (PARP), and β-actin were obtained from Cell Signaling Technology Inc. (Danvers, MA, USA). PGE2 was purchased from Abcam. Membranes were washed with TBS-T buffer and further reacted with secondary antibodies (1:10,000) at 23 °C for 1 h. Membranes were developed using an ECL solution (GenDEPOT, Barker, TX, USA). The intensity of each band was quantified using a LuminoGraph machine manufactured by Atto in Tokyo, Japan. β-actin was utilized as a reference for normalization purposes. The quantification of bands on the membranes was conducted utilizing the ImageJ software.

### 2.8. Statistical Analysis

The data are reported in terms of mean values with standard deviations. Group comparisons were conducted utilizing Student’s *t*-test and one-way analysis of variance where appropriate. Statistical analysis was carried out using Microcal Origin 7 software (Microcal Software Inc., Northampton, MA, USA). Statistical significance was defined as *p* < 0.05 and *p* < 0.01.

## 3. Results

### 3.1. Effect of ACE (AGEprost^®^) on Prostate Development in TP-Induced BPH Rats

**Figure 1:**
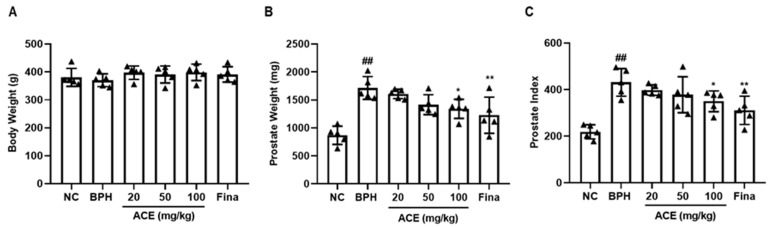
Effect of ACE (AGEprost^®^) treatment on prostate weight and prostate index in the TP-induced BPH rat model. (**A**) Body weight on the last day of treatment, (**B**) prostate weight, and (**C**) prostate index. The values for each animal are presented as a dot plot in the shape of a triangle. prostate index was calculated as follows: prostate weight (mg)/100 g of the body weight. Values = mean ± S.D (*n* = 5). ^##^ *p* < 0.01 vs. NC; * *p* < 0.05, ** *p* < 0.01 vs. BPH. NC: normal control; BPH: benign prostatic hyperplasia; ACE (AGEprost^®^): *Ageratum conyzoides* extract; Fina: finasteride.

The 6 weeks of ACE (AGEprost^®^) administration did not affect the body weight (Figure 1A) and their test article-related clinical chemistry parameters did not differ between the control and experimental groups (Table 1). All biochemical parameters were within the reference range [20]. The BPH groups exhibited a statistically significant elevation of 197.48% in prostate weight when compared to the NC group (*p* < 0.01), and the finasteride-treated group had a significantly reduced prostate weight (28.46%) than that of the BPH group (Figure 1B, *p* < 0.01). The 100 mg/kg ACE (AGEprost^®^)-treated group showed a significant reduction in prostate weight of 21.76% (*p* < 0.05), whereas the 20 mg/kg and 50 mg/kg doses resulted in non-significant reductions of 6.22% and 17.51%, respectively. A comparable pattern was noted in the case of the prostate index. The BPH group showed significantly increased prostatic index than that of the NC group (Figure 1C, *p* < 0.01), whereas the finasteride and ACE (AGEprost^®^)-treated groups showed a decrease in the prostate index relative to that of the BPH group.

**Figure 2:**
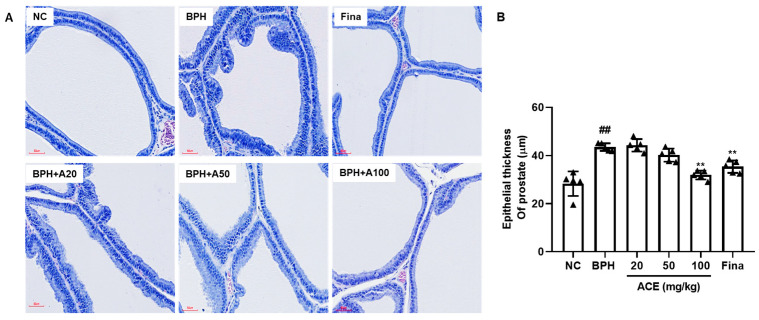
ACE (AGEprost^®^) treatment restored the epithelial thickness of the prostate in the TP-induced BPH rat model. (**A**) Representative H&E staining of prostate tissues, (**B**) the calculated epithelial thickness of the prostate (bar = 60 μm). The values for each animal are presented as a dot plot in the shape of a triangle. Values = mean ± S.D (*n* = 5). ^##^ *p* < 0.01 vs. NC group; ** *p* < 0.01 vs. BPH group. NC: normal control; BPH: benign prostatic hyperplasia; ACE: *Ageratum conyzoides* extract; Fina: finasteride.

In the histological analysis of H&E staining, the BPH group demonstrated a substantial rise in epithelial hyperplasia compared to the NC group. However, the group treated with ACE (AGEprost^®^) at a dosage of 100 mg/kg showed a decrease in levels of epithelial hyperplasia in comparison to the BPH group, similar to the reduction seen in the group treated with finasteride (Figure 2A). Moreover, when compared to the normal control group, the BPH group exhibited a substantial elevation in epithelial thickness within the prostate (ETP) by 154.72% (*p* < 0.01). The orally administered finasteride group significantly restored ETP with a 19.91% inhibition of BPH (Figure 2B, *p* < 0.01). Although the 20 mg and 50 mg/kg ACE (AGEprost^®^)-treated groups showed no significant differences compared to the BPH group, they still exhibited an overall lower thickness than the BPH group. The 100 mg/kg ACE (AGEprost^®^)-treated group significantly restored its epithelial structure with a 27.12% suppression compared to the BPH group (*p* < 0.01).

### 3.2. Effect of ACE (AGEprost^®^) on Androgens and BPH-Related Protein Expression in TP-Induced BPH Rats

**Figure 3:**
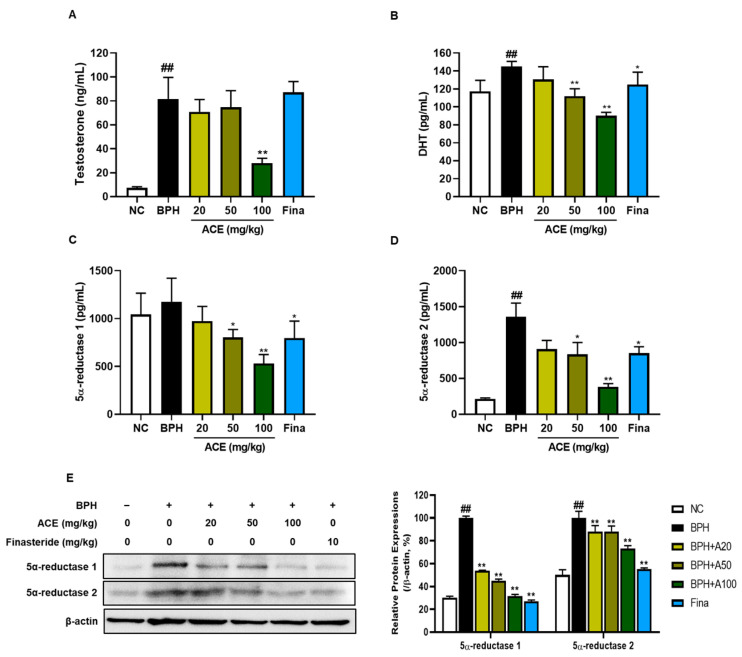
Effects of ACE (AGEprost^®^) treatment on androgen hormones and enzymes. The plasma levels for (**A**) testosterone, (**B**) DHT, (**C**) 5-alpha reductase 1, (**D**) 5-alpha reductase 2, and (**E**) protein expression levels of 5-alpha reductase 1 and 2 determined using Western blotting in the testosterone-induced benign prostate hyperplasia rat model. The loading control utilized in the study was β-actin, and protein expressions were subsequently normalized against this control. Values = mean ± S.D. ^##^ *p* < 0.01 vs. NC group; * *p* < 0.05, ** *p* < 0.01 vs. BPH group. NC: normal control; BPH: benign prostatic hyperplasia; ACE (AGEprost^®^): *Ageratum conyzoides* extract; Fina: finasteride DHT: dihydrotestosterone.

The plasma levels of DHT, testosterone, and 5α-reductase were markedly increased in the BPH group compared to those in the NC group (Figure 3A–D, *p* < 0.01). The levels of these biological molecules decreased dose-dependently in ACE (AGEprost^®^)-treated groups. Specifically, the levels of testosterone, DHT, and 5α-reductase1 and 2 were significantly suppressed in the group treated with 100 mg/kg of standardized ACE by 65.65%, 37.81%, 54.79%, and 71.92%, respectively, compared to those in the BPH group (*p* < 0.01), with no significant difference from the NC group. Finasteride, a known 5α-reductase type 2 inhibitor, significantly suppressed DHT and 5α-reductase type 1 and 2 levels compared to those in the BPH group (*p* < 0.05). Similar to plasma concentrations, the levels of 5α-reductase type 1 and 2 in prostrate tissues showed a significant increase in the BPH group compared to those in the NC group (Figure 3E, *p* < 0.01). Both finasteride and ACE (AGEprost^®^) remarkably attenuated the expression of these enzymes in the prostate relative to those in the BPH group (*p* < 0.01).

### 3.3. Effect of ACE (AGEprost^®^) on Cell Proliferation in TP-Induced BPH Rats

**Figure 4:**
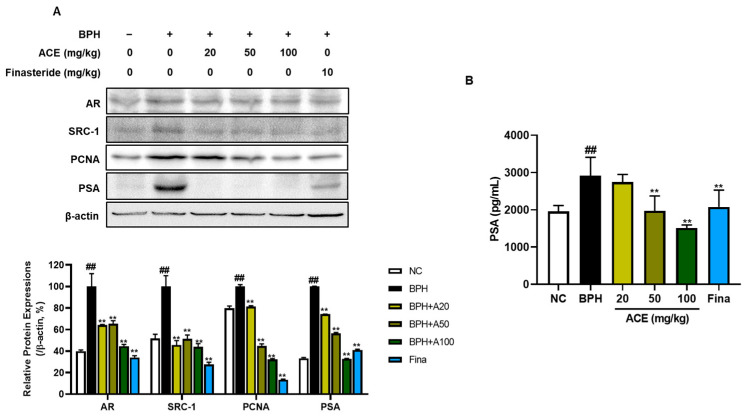
Effects ACE (AGEprost^®^) treatment on androgen receptor- and cell proliferation-related protein expression of prostate in the TP-induced BPH rat model. (**A**) Representative bands and their normalized relative protein expressions of AR, SRC-1, PCNA, and PSA determined using Western blotting. The comparative band intensity ratios of AR, SRC-1, PCNA, and PSA to β-actin in rat prostate tissues. β-actin was used as a loading control. The protein expressions were quantified by measuring its band intensity using the ImageJ software. (**B**) Plasma PSA concentration. Values = mean ± S.D. ^##^ *p* < 0.01 vs. NC group; ** *p* < 0.01 vs. BPH group. NC: normal control; BPH: benign prostatic hyperplasia; ACE (AGEprost^®^): *Ageratum conyzoides* extract; Fina: finasteride.

The stimulation of AR leads to BPH development. We investigated AR-related proliferation markers to evaluate whether ACE (AGEprost^®^) could regulate these markers, which lead to the induction of hyperplasia. The BPH-related proliferation markers AR, SRC-1, PCNA, and PSA were markedly upregulated in the BPH group relative to those in the NC group in both prostate tissues and plasma (Figure 4A, *p* < 0.01). However, finasteride and ACE (AGEprost^®^) significantly suppressed the expression of these proliferation-related proteins. The decrease in protein expressions of AR and SRC-1 were dose-independent in ACE (AGEprost^®^)-treated groups. In the case of PSA, a BPH biomarker, the repression effect of ACE (AGEprost^®^) was more significant than that of finasteride, in both prostate tissues and plasma (Figure 4B, *p* < 0.01).

### 3.4. Effects of ACE (AGEprost^®^) on COX-2/PGE2/Aromatase Cascade in TP-Induced BPH Rats

**Figure 5:**
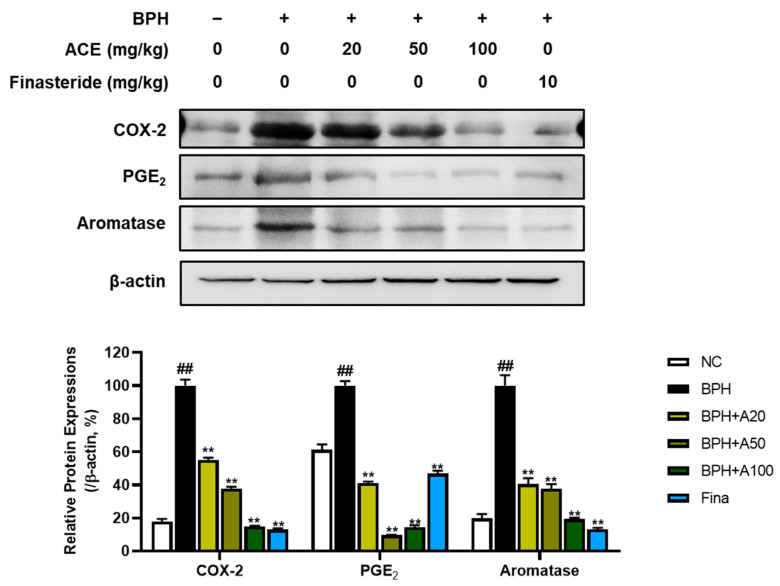
Effects of ACE (AGEprost^®^) treatment on inflammation- and estrogen receptor-related protein expression in the TP-induced BPH rat model. Representative bands and normalized relative protein expressions of COX-2, PGE2, and aromatase in rat prostate tissues were analyzed using Western blotting. β-actin was used as a loading control. The protein expressions were quantified by measuring band intensities using the ImageJ software. Values = mean ± S.D. ^##^ *p* < 0.01 vs. NC group; ** *p* < 0.01 vs. BPH group. NC: normal control; BPH: benign prostatic hyperplasia; ACE (AGEprost^®^): *Ageratum conyzoides* extract; Fina: finasteride.

In the progression of BPH, inflammation also assumes a crucial role. COX-2, PGE2, and Aromatase were examined to explore the suppressive effects of standardized ACE on inflammatory signals. The BPH group demonstrated a notable elevation in the levels of COX-2, PGE2, and aromatase proteins when compared to the NC group (Figure 5, *p* < 0.01). Conversely, treatment with finasteride and ACE markedly decreased the protein levels of these markers (*p* < 0.01).

### 3.5. Effects of ACE (AGEprost^®^) on Apoptosis in TP-Induced BPH Rats

**Figure 6:**
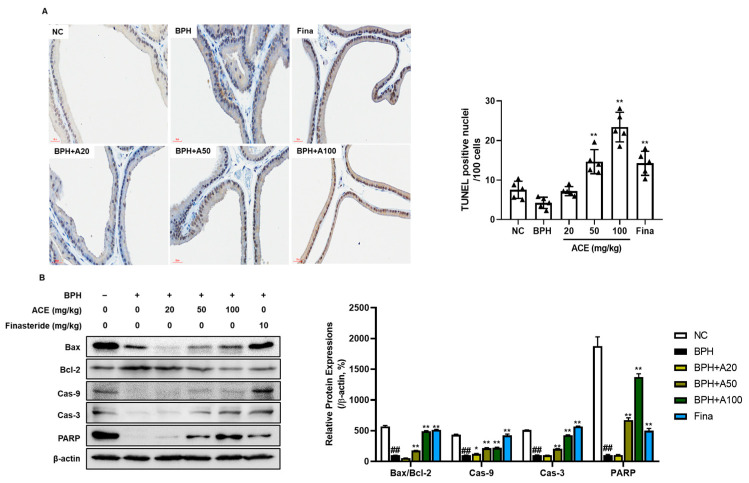
Effects of ACE (AGEprost^®^) treatment on TUNEL-positive cells and apoptosis-related protein expression in the TP-induced BPH rat model (bar = 30 μm). (**A**) Representative photomicrographs of TUNEL-stained prostate tissues of each group (magnification ×400) and the number of TUNEL-positive nuclei per 100 cells. The values for each animal are presented as a dot plot in the shape of a triangle. (**B**) Representative bands and normalized relative protein expressions of Bax, Bcl-2, Cas-9, Cas-3, and PARP in rat prostate tissues were analyzed using Western blotting. β-actin was used as a loading control. The protein expressions were quantified by measuring their band intensity using the ImageJ software. Values = mean ± S.D. ^##^ *p* < 0.01 vs. NC group; * *p* < 0.05, ** *p* < 0.01 vs. BPH group. NC: normal control; BPH: benign prostatic hyperplasia; ACE (AGEprost^®^): *Ageratum conyzoides* extract; Fina: finasteride.

TUNEL staining was performed to evaluate apoptotic cells in the prostate tissue (Figure 6A). In the BPH group, there were fewer TUNEL-positive cells than those in the NC group (*p* < 0.05). However, groups treated with finasteride and ACE (AGEprost^®^) demonstrated a notable increase in the quantity of TUNEL-positive cells (*p* < 0.01). Consistent with TUNEL staining, treatment with finasteride and ACE (AGEprost^®^) increased the ratio of Bax, a proapoptotic protein, to Bcl-2, an anti-apoptotic protein, in the prostate tissue relative to that in the BPH group (Figure 6B, *p* < 0.01). Additionally, downstream proapoptotic cascades, such as caspase 9, caspase 3, and PARP, significantly increased dose-dependently in the ACE (AGEprost^®^)-treated group (*p* < 0.01).

## 4. Discussion

The incidence of BPH has risen with increase in the proportion of the aging population, making it a social concern, due to its detrimental influence on one’s quality of life [21]. As the need for highly efficacious novel BPH therapeutic agents with few adverse effects has intensified, the possibility of using botanical reagents has been explored [22]. This study aimed to investigate the inhibitory effects of orally administered ACE (AGEprost^®^) on BPH in an animal model by assessing pathological, hormonal, inflammatory, and apoptotic factors.

BPH is known as a hormone-dependent disorder, where androgen signaling through its specific receptor is crucial for stimulating the growth of epithelial cells [5]. DHT, synthesized from testosterone by the enzyme 5α-reductase, exhibits higher androgenic potency, stabilizing and activating AR transcriptional activity, thereby promoting the pathogenesis of BPH in men [23,24]. Since DHT is synthesized from testosterone through 5α-reductases, inhibiting 5α-reductases type 2 is a key strategy for treating BPH, which predominantly presents in the hyperplastic glands of the prostate [25,26]. In the current study, TP-induced increases in testosterone and DHT levels were dose-dependently downregulated in ACE (AGEprost^®^)-treated groups in rats. Moreover, the levels of 5α-reductase type 1 and 2 evidently reduced in the ACE (AGEprost^®^)-treated group, with a greater decrease than observed in the finasteride group. This finding was further supported by 5α-reductases’ protein expression. Androgen/AR signaling is a crucial factor in BPH progression [27]. On the binding of androgen to the AR, the complex translocates to the nucleus and associates with co-regulators, such as SRC1. This interaction regulates target gene expression and prostate development processes, such as epithelial cell proliferation, differentiation, and survival [28,29]. In this study, ACE (AGEprost^®^) treatment suppressed AR and SRC-1 expression in the prostate, followed by a decrease in PSA and PCNA expression, which are BPH and prostate proliferation biomarkers, respectively. Therefore, the mechanism by which ACE (AGEprost^®^) alleviates BPH may involve the inhibition of androgens and reductases, subsequently reducing sub-signals associated with prostate proliferation.

*A. conyzoides* is a medicinal plant traditionally used to alleviate inflammation and pain [16]. Several studies have shown that ACE (AGEprost^®^) has anti-inflammatory and analgesic effects by suppressing prostaglandins and inflammatory cytokines [30,31]. Chronic inflammation is linked to BPH progression, potentially involving pro-inflammatory factors like cytokines and prostaglandins [32]. PGE2, a prostanoid synthesized by COX-2, binds to EP4 receptors in the prostate tissue, inducing prostatic inflammation and contributing to lower urinary tract symptoms in BPH [33]. PGE2 also enhances the expression of aromatase, an enzyme that converts testosterone to estrogen in BPH-1 cells [34]. Moreover, recent evidence indicates that estrogen also contributes to the pathogenesis of BPH, with distinct roles for estrogen receptor subtypes ERα and ERβ in prostate cell growth, involving mechanisms like cell death, aromatase activity, and regulation via PGE2 [35]. In the current study, we confirmed the content of the bioactive compound 5′-methoxynobiletin as 0.4% through HPLC analysis. 5′-methoxynobiletin, which is one of the polymethoxyflavones commonly present in *A. conyzoides*, has exhibited anti-inflammatory properties in various scientific investigations. The effectiveness of 5′-methoxynobiletin in alleviating inflammation is linked to its ability to suppress COX-2 and PGE2 [31,36]. Consistent with previous studies, the present study demonstrated that oral administration of ACE (AGEprost^®^) significantly reduced inflammatory factors associated with BPH, such as COX-2, PGE2, and aromatase.

Regulating apoptosis is a therapeutic objective for treating BPH since insufficient apoptotic signals in the prostate during BPH result in the uncontrolled growth of epithelial and stromal cells [37]. Enhanced Bcl-2 expression has been reported in BPH specimens compared to that in normal prostate tissue [38,39]. Consistently, the BPH control group exhibited the highest Bcl-2 expression, accompanied by the inhibition of proapoptotic signals. Meanwhile, ACE (AGEprost^®^)-treated groups showed dose-proportional inhibition of Bcl-2 expression and activated Bax and its downstream apoptotic signals such as Cas-9, Cas-3, and PARP. Furthermore, augmented apoptosis in ACE (AGEprost^®^)-treated groups was confirmed by the increased ratio of TUNEL-positive cells in prostate tissues.

## 5. Conclusions

In conclusion, our findings demonstrated the inhibitory effect of ACE (AGEprost^®^), the ethanolic extract of *A. cornyzoides*, in a BPH rat model. It restored pathological changes associated with BPH by inhibiting androgens, the key factor involved in this phenomenon. Moreover, decreased inflammation and enhanced apoptotic signals were observed after ACE (AGEprost^®^) treatment. These data suggested that ACE may have potential as an herbal medicine for treating BPH. Further investigations into its clinical efficacy and safety in humans, as well as on its active ingredients, are required.

## Figures and Tables

**Table 1:** Effects of ACE (AGEprost^®^) treatment on the blood biochemistry levels (mean ± S.D) in TP-induced benign prostate hyperplasia rat model (*n* = 5).

	AST (u/L)	ALT (u/L)	CRE(mg/dL)	ALB(g/dL)	TG(mg/dL)	TCHO (mg/dL)	BUN (mg/dL)
**NC**	58.00 ± 13.23	102.40 ± 16.34	0.32 ± 0.04	5.07 ± 0.47	96.43 ± 29.55	92.11 ± 33.92	21.51 ± 3.53
**BPH**	88.40 ± 36.31	53.60 ± 25.98	0.32 ± 0.04	5.14 ± 0.24	90.20 ± 22.34	99.40 ± 15.03	26.68 ± 2.74
**Fina**	152.20 ± 93.11	64.60 ± 24.81	0.33 ± 0.07	5.40 ± 0.08	112.80 ± 11.54	116.00 ± 28.42	24.78 ± 3.60
**BPH + A20**	96.88 ± 21.03	47.75 ± 5.99	0.28 ± 0.03	4.81 ± 0.36	93.75 ± 31.60	91.13 ± 15.73	19.98 ± 2.06
**BPH + A50**	110.50 ± 21.53	50.13 ± 4.05	0.34 ± 0.05	4.66 ± 1.24	86.00 ± 33.05	100.50 ± 11.55	22.53 ± 2.19
**BPH + A100**	119.38 ± 24.02	47.38 ± 7.54	0.30 ± 0.04	4.71 ± 0.51	70.63 ± 41.51	96.13 ± 16.57	20.73 ± 4.14

AST: aspartate aminotransferase; ALT: alanine aminotransferase; CRE: creatinine; ALB: albumin; TG: triglyceride; TCHO: total cholesterol; BUN: blood urea nitrogen; NC: normal control; BPH: benign prostatic hyperplasia; ACE: *Ageratum conyzoides* extract; Fina: finasteride.

## Data Availability

The data presented in this study are available on request from the corresponding author due to privacy concerns.

## Supplementary Materials

The following supporting information can be downloaded at https://www.mdpi.com/article/10.3390/nu16142267/s1, Figure S1: High–performance chromatography (HPLC) analysis of ACE (AGEprost^®^). (A) The chromatogram of standardized ACE and (B) the reference standard of 5′-methoxynobiletin. The detection wavelength was 330nm.
