# Supplementary material for: Ageratum conyzoides Extract Ameliorates Testosterone-Induced Benign Prostatic Hyperplasia via Inhibiting Proliferation, Inflammation of Prostates, and Induction of Apoptosis in Rats
Source: Nutrients. 2024 Jul 14;16(14):2267. doi: 10.3390/nu16142267 (PMC11280401; doi:10.3390/nu16142267)
Supplement: Supplementary file 1 [file nutrients-16-02267-s001.zip › nutrients-3102535-supplementary.pdf]

Supplementary Material

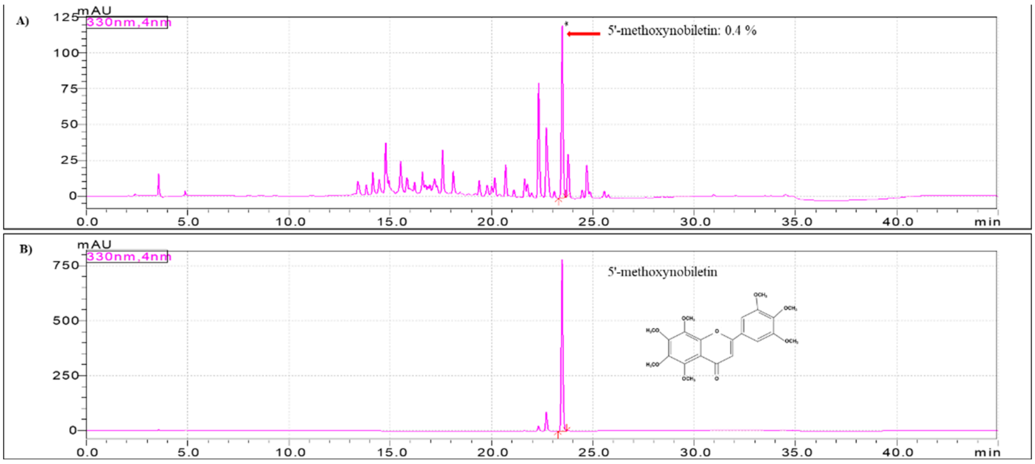

Supplementary Figure S1. High–performance chromatography (HPLC) analysis of ACE (AGEprost®). (A) The chromatogram of standardized ACE and (B) the reference standard of 5′-methoxynobiletin. The detection wavelength was 330nm
